# Supplementary figures and images for: Centromere protein N may be a novel malignant prognostic biomarker for hepatocellular carcinoma
Source: PeerJ. 2021 May 3;9:e11342. doi: 10.7717/peerj.11342 (PMC8101454; doi:10.7717/peerj.11342)

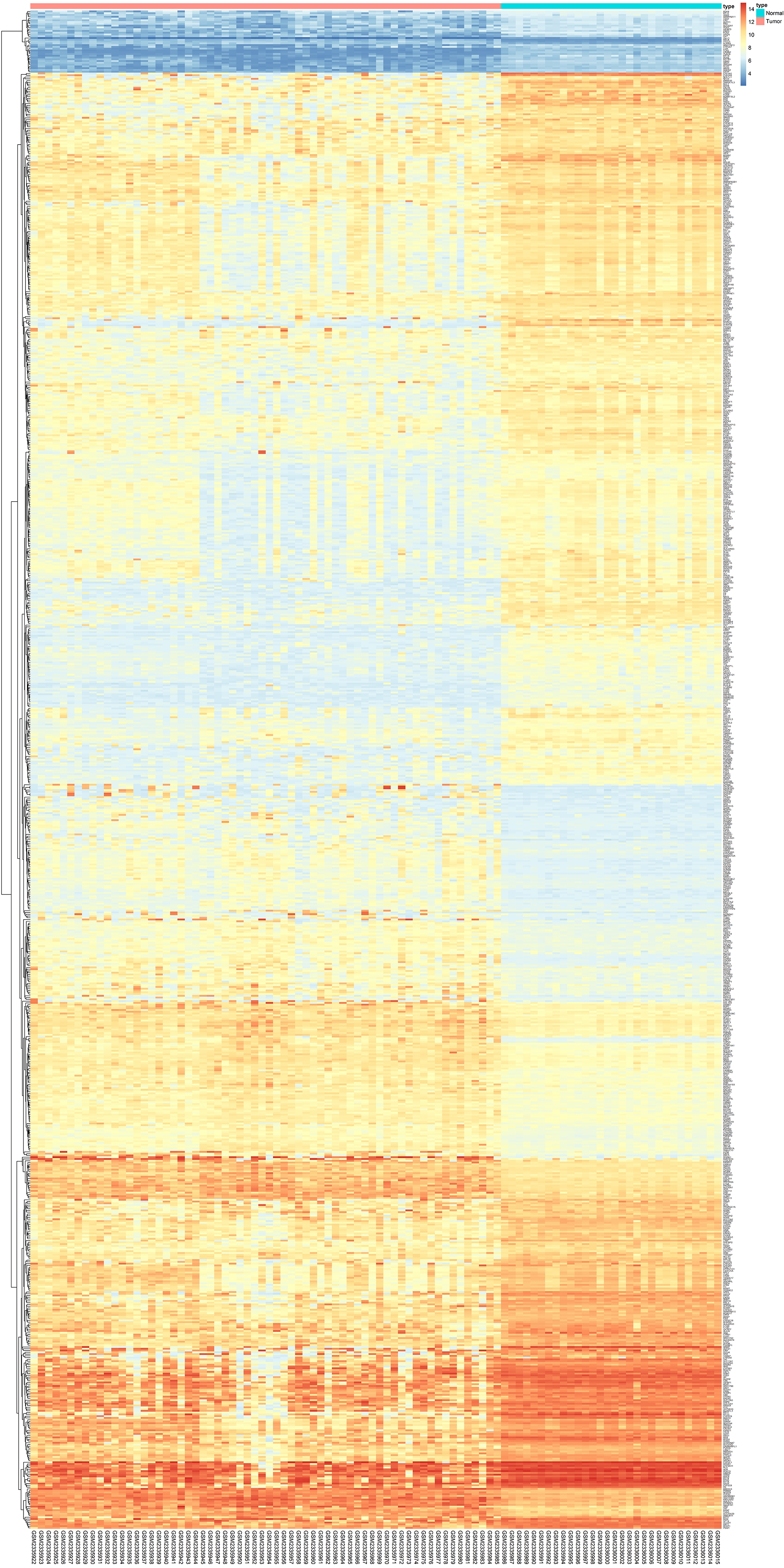

Supplement: Figure S1 — Threshold: FDR < 0.05 and | logFC | > 1. [file peerj-09-11342-s001.png]

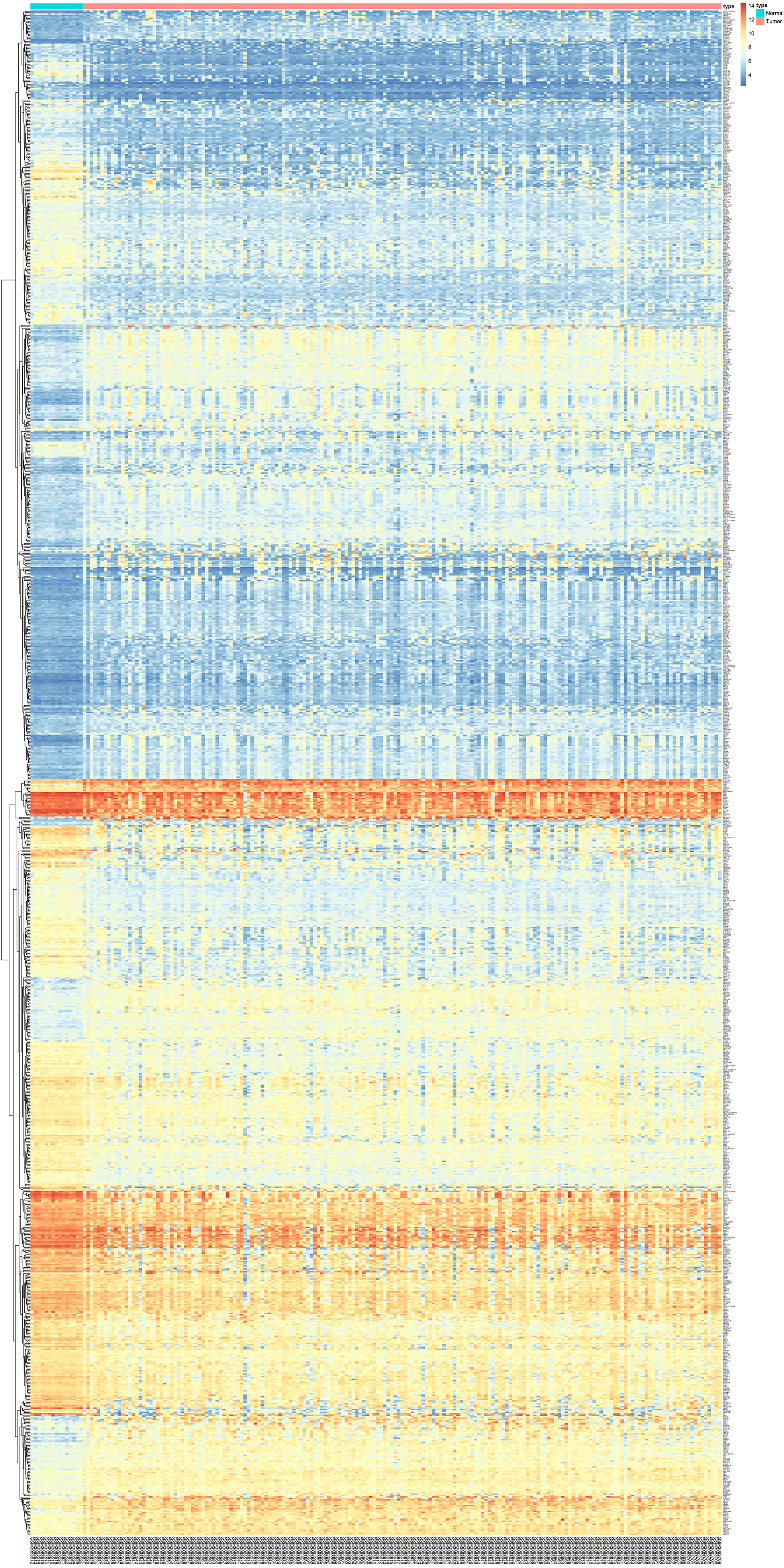

Supplement: Figure S2 — Threshold: FDR < 0.05 and | logFC | > 1. [file peerj-09-11342-s002.png]

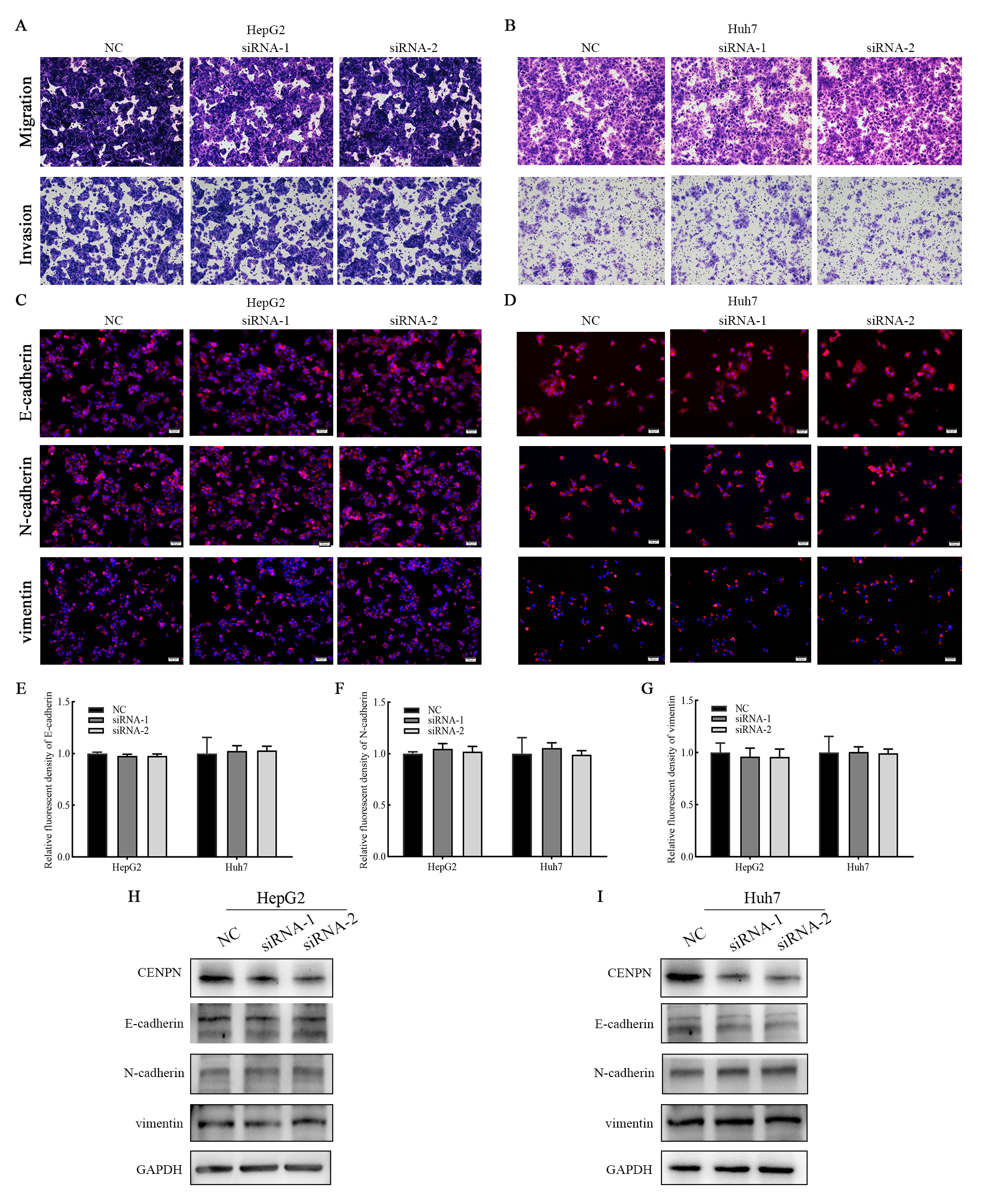

Supplement: Figure S3 — (A, B) Transwell assays were used to detect the number of invasive and migrating HCC cells with CENPN knockdown. (C–G) Immunofluorescence detection of EMT marker expression. (H–I) Western blot assays were performed to detect EMT marker expression in cells with CENPN knockdown. Each data point represents the mean ± SD from three independent experiments. Scale bars, 50 µm. [file peerj-09-11342-s003.png]
