# Supplementary material for: Centromere protein N may be a novel malignant prognostic biomarker for hepatocellular carcinoma
Source: PeerJ. 2021 May 3;9:e11342. doi: 10.7717/peerj.11342 (PMC8101454; doi:10.7717/peerj.11342)
Supplement: Table S1 [file peerj-09-11342-s004.docx]

| Table S1. List of primary antibodies | | | | |
| --- | --- | --- | --- | --- |
| Antigens | Species antibodies raised in | Dilution (IF) | Dilution (WB) | Supplier |
| CENPN | Rabbit | 1:100 | 1:1000 | Affinity, df2315 |
| p53 | Rabbit | - | 1:1000 | Abcam, ab26 |
| p27 | Rabbit | - | 1:1000 | Proteintech, 25614-1-AP |
| p21 | Rabbit | - | 1:1000 | Abcam, ab109520 |
| CDK4 | Rabbit | - | 1:1000 | Abclonal, A0366 |
| cyclinD1 | Rabbit | - | 1:1000 | Proteintech, 60186-1-lg |
| CDK2 | Rabbit | - | 1:1000 | CST, 2546 |
| cyclinE | Rabbit | - | 1:1000 | Abclonal, A14225 |
| pRb (ser780) | Rabbit | - | 1:1000 | CST, 9307 |
| Rb | Rabbit | - | 1:1000 | Proteintech, 25628-1-AP |
| E2F1 | Rabbit | - | 1:1000 | Proteintech, 12171-1-AP |
| c-Myc | Rabbit | - | 1:1000 | Abclonal, A19032 |
| β-tublin  GAPDH | Rabbit  Mouse | -  - | 1:1000  1:5000 | Proteintech, 10094-1-AP  Proteintech, 60004-1-lg |
| ki67 | Rabbit | 1:100 | - | Proteintech, 27309-1-AP |
| γ-H2AX  E-cadherin  N-cadherin  vimentin | Rabbit  Rabbit  Mouse  Rabbit | 1:100  1:100  1:100  1:100 | -  1:1000  1:1000  1:1000 | CST, 2577  Proteintech, 20874-1-AP  Proteintech, 66219-1-Ig  Proteintech, 10366-1-AP |
