# Supplementary material for: Centromere protein N may be a novel malignant prognostic biomarker for hepatocellular carcinoma
Source: PeerJ. 2021 May 3;9:e11342. doi: 10.7717/peerj.11342 (PMC8101454; doi:10.7717/peerj.11342)
Supplement: Table S2 [file peerj-09-11342-s005.docx]

| Table S2. The overlapping DEGs between GSE87630 and GSE112790 | | |
| --- | --- | --- |
| Names | Total | Elements |
| Both Up | 171 | PRC1 TOP2A PTTG1 KIF20A CDC20 PLVAP CDCA8 KRTCAP2 CDCA5 MELK RACGAP1 SCAMP3 AURKA TBC1D31 CCNB2 HJURP FEN1 NUP62 NUSAP1 CENPF MCM4 UBE2C TUBG1 FLAD1 FAM189B TK1 TRIP13 CCDC34 VPS72 CKAP5 KIF4A AURKB SETDB1 PLOD3 RRAGC CAP2 MCM2 NCAPD2 LSM4 TTC13 KIF2C HMMR OXLD1 MPV17 ZNF260 COG2 PPOX STMN1 MCM6 FOXK1 TKT NSMCE2 C1orf112 MDK CTSA SNRPB CENPM AKR1C3 FOXM1 UBE2Q1 GPSM2 EIF2D SAC3D1 TPX2 FAM83H COL4A1 OIP5 GLMP PEA15 CASP2 AP3M2 SF3B4 TLCD1 NEU1 SCRIB CKAP4 IGSF3 KNTC1 CLN3 MCM5 UHRF1 CCNA2 FAM83D ANXA2 NECAB3 MYO5C ATAD2 PRCC SLC52A2 RNASEH2A GLA PARP12 RUSC1 ANO10 BARD1 GPC3 TMEM9 ZIC2 THY1 SPINK1 UBE2T HSPB1 PIEZO2 TMEM106C TAGLN2 TP53I3 STK39 MCM7 OLFML2B MCM3 CENPN CDKN3 SPNS1 POLE2 TARBP1 NOTCH3 PAFAH1B3 TUG1 G6PD TOR3A FKBP11 NCAPG TXNRD1 AKR1B10 DTNA GINS2 GBP2 HRCT1 SRXN1 TMEM45B CEBPA RRAGD NEDD4L SQLE ASPM MRAP2 SULT1C2 SORT1 NT5DC2 COL1A1 SFN ZNF704 STC2 C1orf198 MUC13 MICB SLC2A5 SSUH2 HKDC1 VWF NUPR1 S100P MAGEA1 COX7B2 LCN2 SLC51B ASNS NQO1 SSX1 IGF2BP2 IGF2BP3 TRNP1 CA12 CCL20 LGALS4 EEF1A2 LAPTM4B REG3A PAGE4 ALDH3A1 SPP1 |
| Both Down | 361 | STAB2 CLEC4G CLEC1B ECM1 CRHBP KBTBD11 CLEC4M FCN2 ADGRG7 INMT FOS CFP MARCO CXCL14 C7 GYS2 COLEC10 DEPDC7 SRPX LYVE1 NAT2 PLAC8 OIT3 ANGPTL6 FCN3 ANKRD55 VIPR1 N4BP2L1 CXCL2 PTH1R GHR MT1M SOCS2 CXCL12 SLCO1B3 RND3 DNASE1L3 SKAP1 CDHR2 SLC38A4 CLRN3 JCHAIN KMO FAM134B CETP ADAMTS1 SRD5A2 MT1F EGR2 CYP1A2 FOSB PTGS2 RCAN1 TTC36 MT1H ESR1 PDGFRA NAAA BCHE ACSL1 C9 PANK1 CD4 GBA3 IGFALS MS4A6A ZFP36L1 CPED1 IGFBP3 ALDH8A1 KDM8 AADAT EPB41L4B C11orf96 HAO1 SLCO1B1 SLC38A2 PTP4A1 AGXT2 RHOB SLC25A47 ZFP36 TIMD4 NFIL3 C8orf4 MPC1 HBB HAMP LCAT DCN F9 KCNN2 ID2 TMEM27 SPRY2 FYN IL13RA2 ABAT CSAD ACSM3 PLSCR4 CD163 IL1RAP DMGDH ADK ENPEP IYD CNDP1 CFI ASS1 RBP5 ACAA2 MAP2K1 NADK2 NNMT CYP39A1 GNE SLC19A3 CYR61 FAM13A ATF3 CYP3A4 HGFAC MYO1B ID1 CTH CYP26A1 FXYD1 ETFDH CLDN10 GCH1 MAN1C1 GLS2 PDE7B MCL1 AGTR1 ANO1 IL33 NR4A2 PEMT IDO2 CYP2C9 ANXA10 RCL1 TACSTD2 LY6E GADD45G MSRA OLFML3 EPHX2 FHL2 HOGA1 CP COLEC11 SMIM24 MBNL2 SAT1 HAO2 ST3GAL6 MT1E DUSP16 CA2 IDNK EGR1 LDLR NCOR1 MT1G HLF C1RL CIDEB JUNB GCDH PHLDA1 BBOX1 FOXO1 PDK4 GPD1 ACADM ADH1B IRF8 TKFC HAL KLKB1 CPT2 CYP2C19 PLIN2 SLC22A1 AFM ADH4 ZG16 MYC TDO2 ECM2 APOF GCKR SPP2 ALDH2 MT1X NDRG2 CPEB3 MTTP CLYBL THBS1 GLYAT TMEM45A RNF125 MARC2 LEPR PROZ SLC7A2 ABCA8 MT2A ADH6 ACADS TENM1 GABARAPL1 MFSD2A ADH1C NAPSB RORA ENO3 DUSP1 GJB2 CNGA1 CYP3A43 CFHR3 GADD45B LIPC TRPM8 WDR72 MBL2 CSRNP1 TAT CYP2J2 CYP2B6 CYP2C8 CITED2 C1orf168 CYP4V2 ACAT1 DUSP6 F11 FBP1 AMDHD1 OAT XDH TBX15 DNAJC12 CYP4F2 GPAT3 MLYCD HSD17B2 STEAP3 FETUB TMEM56 LDHD ATOH8 SHBG CYP2C18 CAT RDH16 CYP4F3 PCK1 AKR7A3 C8A PZP CDO1 S100A8 EVA1A SERPINA10 RGN HPX SLC39A5 GOT1 ARG1 C8B RIDA CPS1 DUSP5 C6 CDA FAM149A SLC17A2 SLC27A5 CYP3A5 SLC27A2 FMO3 LECT2 ALDH6A1 ANKRD37 SERPINE1 PON1 ADCY1 CYP4A11 GREM2 PBLD ANGPTL4 NOCT ADH1A ETS2 SLC10A1 ACMSD SLC46A3 ST6GAL1 AZGP1 CYP2A7 G6PC GSPT2 EHHADH CYP2E1 ACOT12 RNASE4 SLC25A15 CFHR4 SIK1 GCGR HPD APOA5 LPA IGFBP1 SDS HABP2 PPARGC1A GNMT THRSP HSD11B1 ACSM5 CPN1 ALPL HSD17B6 AOX1 PLG VNN1 BHMT FTCD PXMP2 C1R APCS ANG CYP2A6 GPT2 CYP4F12 PRODH2 OGDHL NR1I3 PCOLCE HPR ATF5 ABCB4 OTC NPC1L1 BGN CYP8B1 SLC25A18 RELN ALDOB HPGD CRP SLC13A5 PPP1R1A ALDH1L1 |
