# Supplementary material for: Centromere protein N may be a novel malignant prognostic biomarker for hepatocellular carcinoma
Source: PeerJ. 2021 May 3;9:e11342. doi: 10.7717/peerj.11342 (PMC8101454; doi:10.7717/peerj.11342)
Supplement: Table S3 [file peerj-09-11342-s006.docx]

| Table S3. The GO enrichment analysis of DEGs | | | | | | | | | |
| --- | --- | --- | --- | --- | --- | --- | --- | --- | --- |
| ONTOLOGY | ID | Description | GeneRatio | BgRatio | pvalue | p.adjust | qvalue | geneID | Count |
| BP | GO:0016054 | organic acid catabolic process | 54/498 | 282/18866 | 5.76E-31 | 1.26E-27 | 1.04E-27 | HMMR/STAB2/LYVE1/KMO/ALDH8A1/AADAT/HAO1/AGXT2/ABAT/CSAD/ACAA2/CTH/CYP26A1/ETFDH/GLS2/IDO2/HOGA1/HAO2/IDNK/GCDH/ACADM/HAL/CPT2/TDO2/ACADS/MFSD2A/TAT/ACAT1/AMDHD1/OAT/CYP4F2/MLYCD/LDHD/CYP4F3/PCK1/CDO1/GOT1/ARG1/RIDA/SLC27A2/ALDH6A1/PON1/CYP4A11/ACMSD/EHHADH/HPD/SDS/FTCD/GPT2/CYP4F12/PRODH2/OTC/HPGD/ALDH1L1 | 54 |
| BP | GO:0046395 | carboxylic acid catabolic process | 54/498 | 282/18866 | 5.76E-31 | 1.26E-27 | 1.04E-27 | HMMR/STAB2/LYVE1/KMO/ALDH8A1/AADAT/HAO1/AGXT2/ABAT/CSAD/ACAA2/CTH/CYP26A1/ETFDH/GLS2/IDO2/HOGA1/HAO2/IDNK/GCDH/ACADM/HAL/CPT2/TDO2/ACADS/MFSD2A/TAT/ACAT1/AMDHD1/OAT/CYP4F2/MLYCD/LDHD/CYP4F3/PCK1/CDO1/GOT1/ARG1/RIDA/SLC27A2/ALDH6A1/PON1/CYP4A11/ACMSD/EHHADH/HPD/SDS/FTCD/GPT2/CYP4F12/PRODH2/OTC/HPGD/ALDH1L1 | 54 |
| BP | GO:0044282 | small molecule catabolic process | 64/498 | 452/18866 | 1.17E-28 | 1.71E-25 | 1.41E-25 | HMMR/FOXK1/AKR1C3/AKR1B10/STAB2/LYVE1/KMO/ALDH8A1/AADAT/HAO1/AGXT2/ABAT/CSAD/ACAA2/CYP39A1/CTH/CYP26A1/ETFDH/GLS2/IDO2/HOGA1/HAO2/IDNK/GCDH/ACADM/TKFC/HAL/CPT2/ADH4/TDO2/ALDH2/ACADS/MFSD2A/ENO3/TAT/ACAT1/AMDHD1/OAT/CYP4F2/MLYCD/LDHD/CYP4F3/PCK1/CDO1/GOT1/ARG1/RIDA/CDA/SLC27A2/ALDH6A1/PON1/CYP4A11/ACMSD/EHHADH/HPD/SDS/FTCD/GPT2/CYP4F12/PRODH2/OTC/ALDOB/HPGD/ALDH1L1 | 64 |
| BP | GO:1901605 | alpha-amino acid metabolic process | 39/498 | 202/18866 | 9.55E-23 | 1.04E-19 | 8.60E-20 | PLOD3/CLN3/ASNS/KMO/ALDH8A1/AADAT/AGXT2/CSAD/IYD/ASS1/CTH/GLS2/PEMT/IDO2/MSRA/HOGA1/GCDH/HAL/TDO2/GLYAT/TAT/ACAT1/AMDHD1/OAT/CDO1/GOT1/ARG1/RIDA/CPS1/ALDH6A1/ACMSD/HPD/SDS/GNMT/BHMT/FTCD/GPT2/PRODH2/OTC | 39 |
| BP | GO:0008202 | steroid metabolic process | 46/498 | 332/18866 | 2.11E-20 | 1.84E-17 | 1.52E-17 | AKR1C3/G6PD/CEBPA/SQLE/SPP1/CETP/SRD5A2/CYP1A2/ESR1/PDGFRA/LCAT/ACAA2/CYP39A1/CYP3A4/CYP26A1/AGTR1/CYP2C9/EPHX2/EGR1/LDLR/CYP2C19/APOF/LEPR/RORA/CYP3A43/LIPC/CYP2B6/CYP2C8/CYP4V2/ACAT1/HSD17B2/CAT/RDH16/SLC27A5/CYP3A5/SLC27A2/PON1/G6PC/CYP2E1/APOA5/PPARGC1A/HSD11B1/HSD17B6/CYP2A6/NPC1L1/CYP8B1 | 46 |
| BP | GO:1901606 | alpha-amino acid catabolic process | 27/498 | 104/18866 | 1.19E-19 | 7.59E-17 | 6.25E-17 | KMO/ALDH8A1/AADAT/AGXT2/CSAD/GLS2/IDO2/HOGA1/GCDH/HAL/TDO2/TAT/ACAT1/AMDHD1/OAT/CDO1/GOT1/ARG1/RIDA/ALDH6A1/ACMSD/HPD/SDS/FTCD/GPT2/PRODH2/OTC | 27 |
| BP | GO:0009063 | cellular amino acid catabolic process | 29/498 | 124/18866 | 1.21E-19 | 7.59E-17 | 6.25E-17 | KMO/ALDH8A1/AADAT/AGXT2/ABAT/CSAD/CTH/GLS2/IDO2/HOGA1/GCDH/HAL/TDO2/TAT/ACAT1/AMDHD1/OAT/CDO1/GOT1/ARG1/RIDA/ALDH6A1/ACMSD/HPD/SDS/FTCD/GPT2/PRODH2/OTC | 29 |
| BP | GO:0006631 | fatty acid metabolic process | 48/498 | 396/18866 | 8.73E-19 | 4.78E-16 | 3.93E-16 | AKR1C3/CYP1A2/PTGS2/NAAA/ACSL1/HAO1/ACSM3/ACAA2/CYP3A4/ETFDH/CYP2C9/EPHX2/HAO2/NCOR1/GCDH/PDK4/ACADM/CPT2/CYP2C19/ADH4/ACADS/MFSD2A/LIPC/CYP2J2/CYP2B6/CYP2C8/CYP4V2/ACAT1/CYP4F2/MLYCD/CYP2C18/CYP4F3/PCK1/RGN/SLC27A5/SLC27A2/PON1/CYP4A11/CYP2A7/EHHADH/CYP2E1/ACOT12/APOA5/PPARGC1A/ACSM5/CYP2A6/CYP4F12/HPGD | 48 |
| BP | GO:0019373 | epoxygenase P450 pathway | 14/498 | 20/18866 | 2.23E-18 | 1.09E-15 | 8.94E-16 | CYP1A2/CYP2C9/EPHX2/CYP2C19/CYP2J2/CYP2B6/CYP2C8/CYP4F2/CYP2C18/CYP4A11/CYP2A7/CYP2E1/CYP2A6/CYP4F12 | 14 |
| BP | GO:0016053 | organic acid biosynthetic process | 45/498 | 368/18866 | 8.04E-18 | 3.52E-15 | 2.90E-15 | PLOD3/AKR1C3/CLN3/ASNS/KMO/CYP1A2/PTGS2/ALDH8A1/AGXT2/DCN/ABAT/CSAD/ACSM3/ASS1/CYP39A1/CYP3A4/CTH/GCH1/GLS2/CYP2C9/EPHX2/HOGA1/PDK4/LIPC/CYP2C8/OAT/MLYCD/CDO1/RGN/GOT1/CPS1/SLC27A5/SLC27A2/CYP4A11/ACMSD/CYP2E1/APOA5/SDS/ACSM5/BHMT/GPT2/OTC/BGN/CYP8B1/HPGD | 45 |
| CC | GO:0062023 | collagen-containing extracellular matrix | 34/511 | 427/19559 | 9.19E-09 | 2.29E-06 | 2.10E-06 | PLOD3/MDK/COL4A1/ANXA2/GPC3/COL1A1/VWF/LGALS4/ECM1/FCN2/CFP/SRPX/ANGPTL6/FCN3/CXCL12/ADAMTS1/DCN/F9/ZG16/ECM2/SPP2/THBS1/MBL2/PZP/S100A8/HPX/SERPINE1/ANGPTL4/AZGP1/PLG/APCS/ANG/PCOLCE/BGN | 34 |
| CC | GO:0072562 | blood microparticle | 19/511 | 148/19559 | 1.13E-08 | 2.29E-06 | 2.10E-06 | FCN2/FCN3/JCHAIN/BCHE/C9/HBB/CP/C1RL/AFM/CFHR3/C8A/PZP/HPX/PON1/ANGPTL4/PLG/C1R/APCS/HPR | 19 |
| CC | GO:0042555 | MCM complex | 6/511 | 12/19559 | 2.50E-07 | 2.16E-05 | 1.97E-05 | MCM4/MCM2/MCM6/MCM5/MCM7/MCM3 | 6 |
| CC | GO:0034358 | plasma lipoprotein particle | 9/511 | 36/19559 | 2.65E-07 | 2.16E-05 | 1.97E-05 | CETP/LCAT/LDLR/APOF/LIPC/PON1/APOA5/LPA/HPR | 9 |
| CC | GO:1990777 | lipoprotein particle | 9/511 | 36/19559 | 2.65E-07 | 2.16E-05 | 1.97E-05 | CETP/LCAT/LDLR/APOF/LIPC/PON1/APOA5/LPA/HPR | 9 |
| CC | GO:0032994 | protein-lipid complex | 9/511 | 39/19559 | 5.57E-07 | 3.53E-05 | 3.23E-05 | CETP/LCAT/LDLR/APOF/LIPC/PON1/APOA5/LPA/HPR | 9 |
| CC | GO:0005788 | endoplasmic reticulum lumen | 25/511 | 308/19559 | 6.09E-07 | 3.53E-05 | 3.23E-05 | PLOD3/COL4A1/CKAP4/GPC3/TOR3A/COL1A1/STC2/SPP1/CFP/PTGS2/BCHE/CD4/IGFBP3/F9/CP/SPP2/MTTP/THBS1/PROZ/LIPC/EVA1A/SERPINA10/SLC27A2/APOA5/IGFBP1 | 25 |
| CC | GO:0034364 | high-density lipoprotein particle | 7/511 | 27/19559 | 4.50E-06 | 0.000228 | 0.000209 | CETP/LCAT/APOF/LIPC/PON1/APOA5/HPR | 7 |
| CC | GO:0005777 | peroxisome | 13/511 | 137/19559 | 6.35E-05 | 0.002579 | 0.00236 | MPV17/TKT/ACSL1/HAO1/EPHX2/HAO2/XDH/MLYCD/CAT/RIDA/SLC27A2/EHHADH/PXMP2 | 13 |
| CC | GO:0042579 | microbody | 13/511 | 137/19559 | 6.35E-05 | 0.002579 | 0.00236 | MPV17/TKT/ACSL1/HAO1/EPHX2/HAO2/XDH/MLYCD/CAT/RIDA/SLC27A2/EHHADH/PXMP2 | 13 |
| MF | GO:0016712 | oxidoreductase activity, acting on paired donors, with incorporation or reduction of molecular oxygen, reduced flavin or flavoprotein as one donor, and incorporation of one atom of oxygen | 17/500 | 35/18352 | 5.54E-18 | 4.08E-15 | 3.31E-15 | CYP1A2/CYP3A4/CYP2C9/CYP2C19/CYP3A43/CYP2J2/CYP2B6/CYP2C8/CYP4F2/CYP2C18/CYP4F3/CYP3A5/CYP4A11/CYP2A7/CYP2E1/CYP2A6/CYP4F12 | 17 |
| MF | GO:0004497 | monooxygenase activity | 25/500 | 101/18352 | 1.99E-17 | 7.33E-15 | 5.95E-15 | AKR1C3/SQLE/KMO/CYP1A2/CYP39A1/CYP3A4/CYP26A1/CYP2C9/CYP2C19/CYP3A43/CYP2J2/CYP2B6/CYP2C8/CYP4V2/CYP4F2/CYP2C18/CYP4F3/CYP3A5/FMO3/CYP4A11/CYP2A7/CYP2E1/CYP2A6/CYP4F12/CYP8B1 | 25 |
| MF | GO:0008392 | arachidonic acid epoxygenase activity | 12/500 | 16/18352 | 2.42E-16 | 4.68E-14 | 3.80E-14 | CYP2C9/CYP2C19/CYP2J2/CYP2B6/CYP2C8/CYP4F2/CYP2C18/CYP4A11/CYP2A7/CYP2E1/CYP2A6/CYP4F12 | 12 |
| MF | GO:0008391 | arachidonic acid monooxygenase activity | 13/500 | 20/18352 | 2.55E-16 | 4.68E-14 | 3.80E-14 | CYP2C9/CYP2C19/CYP2J2/CYP2B6/CYP2C8/CYP4F2/CYP2C18/CYP4F3/CYP4A11/CYP2A7/CYP2E1/CYP2A6/CYP4F12 | 13 |
| MF | GO:0070330 | aromatase activity | 14/500 | 25/18352 | 3.51E-16 | 5.17E-14 | 4.20E-14 | CYP1A2/CYP3A4/CYP2C9/CYP2C19/CYP3A43/CYP2C8/CYP4F2/CYP2C18/CYP4F3/CYP3A5/CYP4A11/CYP2A7/CYP2E1/CYP4F12 | 14 |
| MF | GO:0016705 | oxidoreductase activity, acting on paired donors, with incorporation or reduction of molecular oxygen | 29/500 | 162/18352 | 6.15E-16 | 6.50E-14 | 5.28E-14 | PLOD3/AKR1C3/SQLE/KMO/CYP1A2/PTGS2/KDM8/CYP39A1/CYP3A4/CYP26A1/CYP2C9/BBOX1/CYP2C19/CYP3A43/CYP2J2/CYP2B6/CYP2C8/CYP4V2/CYP4F2/CYP2C18/CYP4F3/CYP3A5/FMO3/CYP4A11/CYP2A7/CYP2E1/CYP2A6/CYP4F12/CYP8B1 | 29 |
| MF | GO:0020037 | heme binding | 27/500 | 138/18352 | 6.18E-16 | 6.50E-14 | 5.28E-14 | STC2/CYP1A2/PTGS2/HBB/CYP39A1/CYP3A4/CYP26A1/IDO2/CYP2C9/CYP2C19/TDO2/CYP3A43/CYP2J2/CYP2B6/CYP2C8/CYP4V2/CYP4F2/CYP2C18/CAT/CYP4F3/CYP3A5/CYP4A11/CYP2A7/CYP2E1/CYP2A6/CYP4F12/CYP8B1 | 27 |
| MF | GO:0046906 | tetrapyrrole binding | 27/500 | 148/18352 | 3.87E-15 | 3.56E-13 | 2.89E-13 | STC2/CYP1A2/PTGS2/HBB/CYP39A1/CYP3A4/CYP26A1/IDO2/CYP2C9/CYP2C19/TDO2/CYP3A43/CYP2J2/CYP2B6/CYP2C8/CYP4V2/CYP4F2/CYP2C18/CAT/CYP4F3/CYP3A5/CYP4A11/CYP2A7/CYP2E1/CYP2A6/CYP4F12/CYP8B1 | 27 |
| MF | GO:0005506 | iron ion binding | 27/500 | 151/18352 | 6.50E-15 | 5.31E-13 | 4.32E-13 | PLOD3/LCN2/CYP1A2/CYP39A1/CYP3A4/CYP26A1/CYP2C9/BBOX1/CYP2C19/CYP3A43/CYP2J2/CYP2B6/CYP2C8/CYP4V2/XDH/CYP4F2/CYP2C18/CYP4F3/CDO1/CYP3A5/CYP4A11/CYP2A7/CYP2E1/AOX1/CYP2A6/CYP4F12/CYP8B1 | 27 |
| MF | GO:0008395 | steroid hydroxylase activity | 15/500 | 38/18352 | 2.39E-14 | 1.76E-12 | 1.43E-12 | CYP1A2/CYP39A1/CYP3A4/CYP2C9/CYP2C19/CYP3A43/CYP2J2/CYP2B6/CYP2C8/CYP2C18/CYP3A5/CYP2A7/CYP2E1/CYP2A6/CYP8B1 | 15 |
