# Supplementary material for: Centromere protein N may be a novel malignant prognostic biomarker for hepatocellular carcinoma
Source: PeerJ. 2021 May 3;9:e11342. doi: 10.7717/peerj.11342 (PMC8101454; doi:10.7717/peerj.11342)
Supplement: Table S4 [file peerj-09-11342-s007.docx]

| Table S4. The KEGG enrichment analysis of DEGs | | | | | | | | |
| --- | --- | --- | --- | --- | --- | --- | --- | --- |
| ID | Description | GeneRatio | BgRatio | pvalue | p.adjust | qvalue | geneID | Count |
| hsa05204 | Chemical carcinogenesis | 20/329 | 83/8101 | 6.67E-11 | 1.65E-08 | 1.42E-08 | ALDH3A1/NAT2/CYP1A2/PTGS2/CYP3A4/CYP2C9/ADH1B/CYP2C19/ADH4/ADH6/ADH1C/CYP3A43/CYP2C8/CYP2C18/CYP3A5/ADH1A/CYP2A7/CYP2E1/HSD11B1/CYP2A6 | 20 |
| hsa00830 | Retinol metabolism | 18/329 | 68/8101 | 1.14E-10 | 1.65E-08 | 1.42E-08 | CYP1A2/CYP3A4/CYP26A1/CYP2C9/ADH1B/ADH4/ADH6/ADH1C/CYP2B6/CYP2C8/CYP2C18/RDH16/CYP3A5/ADH1A/CYP2A7/HSD17B6/AOX1/CYP2A6 | 18 |
| hsa00982 | Drug metabolism - cytochrome P450 | 18/329 | 72/8101 | 3.20E-10 | 3.08E-08 | 2.65E-08 | ALDH3A1/CYP1A2/CYP3A4/CYP2C9/ADH1B/CYP2C19/ADH4/ADH6/ADH1C/CYP2B6/CYP2C8/CYP3A5/FMO3/ADH1A/CYP2A7/CYP2E1/AOX1/CYP2A6 | 18 |
| hsa00380 | Tryptophan metabolism | 14/329 | 42/8101 | 4.76E-10 | 3.44E-08 | 2.96E-08 | INMT/KMO/CYP1A2/ALDH8A1/AADAT/IDO2/GCDH/TDO2/ALDH2/ACAT1/CAT/ACMSD/EHHADH/AOX1 | 14 |
| hsa00071 | Fatty acid degradation | 14/329 | 43/8101 | 6.80E-10 | 3.93E-08 | 3.38E-08 | ACSL1/ACAA2/GCDH/ACADM/ADH1B/CPT2/ADH4/ALDH2/ADH6/ACADS/ADH1C/ACAT1/ADH1A/EHHADH | 14 |
| hsa01200 | Carbon metabolism | 21/329 | 118/8101 | 8.42E-09 | 4.06E-07 | 3.49E-07 | TKT/G6PD/HKDC1/HAO1/HAO2/IDNK/TKFC/ACADS/ENO3/ACAT1/FBP1/CAT/RGN/GOT1/CPS1/ALDH6A1/EHHADH/SDS/GPT2/OGDHL/ALDOB | 21 |
| hsa00980 | Metabolism of xenobiotics by cytochrome P450 | 16/329 | 78/8101 | 6.62E-08 | 2.73E-06 | 2.35E-06 | ALDH3A1/CYP1A2/CYP3A4/CYP2C9/ADH1B/ADH4/ADH6/ADH1C/CYP2B6/AKR7A3/CYP3A5/ADH1A/CYP2A7/CYP2E1/HSD11B1/CYP2A6 | 16 |
| hsa04610 | Complement and coagulation cascades | 16/329 | 85/8101 | 2.34E-07 | 8.44E-06 | 7.26E-06 | VWF/C7/C9/F9/CFI/KLKB1/CFHR3/MBL2/F11/C8A/C8B/C6/SERPINE1/CFHR4/PLG/C1R | 16 |
| hsa00350 | Tyrosine metabolism | 10/329 | 36/8101 | 1.05E-06 | 3.39E-05 | 2.91E-05 | ALDH3A1/ADH1B/ADH4/ADH6/ADH1C/TAT/GOT1/ADH1A/HPD/AOX1 | 10 |
| hsa00010 | Glycolysis / Gluconeogenesis | 13/329 | 67/8101 | 2.25E-06 | 6.51E-05 | 5.59E-05 | HKDC1/ALDH3A1/ADH1B/ADH4/ALDH2/ADH6/ADH1C/ENO3/FBP1/PCK1/ADH1A/G6PC/ALDOB | 13 |
| hsa03030 | DNA replication | 9/329 | 36/8101 | 9.60E-06 | 0.00025225 | 0.00021683 | FEN1/MCM4/MCM2/MCM6/MCM5/RNASEH2A/MCM7/MCM3/POLE2 | 9 |
| hsa00620 | Pyruvate metabolism | 10/329 | 47/8101 | 1.44E-05 | 0.00034731 | 0.00029854 | ADH1B/ADH4/ALDH2/ADH6/ADH1C/ACAT1/LDHD/PCK1/ADH1A/ACOT12 | 10 |
| hsa00220 | Arginine biosynthesis | 7/329 | 22/8101 | 1.72E-05 | 0.00035002 | 0.00030087 | ASS1/GLS2/GOT1/ARG1/CPS1/GPT2/OTC | 7 |
| hsa00280 | Valine, leucine and isoleucine degradation | 10/329 | 48/8101 | 1.76E-05 | 0.00035002 | 0.00030087 | AGXT2/ABAT/ACAA2/ACADM/ALDH2/ACADS/ACAT1/ALDH6A1/EHHADH/AOX1 | 10 |
| hsa00410 | beta-Alanine metabolism | 8/329 | 30/8101 | 1.82E-05 | 0.00035002 | 0.00030087 | ALDH3A1/ABAT/CNDP1/ALDH2/ACADS/MLYCD/ALDH6A1/EHHADH | 8 |
| hsa00590 | Arachidonic acid metabolism | 11/329 | 61/8101 | 2.84E-05 | 0.00051214 | 0.00044023 | AKR1C3/PTGS2/CYP2C9/EPHX2/CYP2C19/CYP2J2/CYP2B6/CYP2C8/CYP4F2/CYP4F3/CYP2E1 | 11 |
| hsa01230 | Biosynthesis of amino acids | 12/329 | 75/8101 | 4.29E-05 | 0.00072936 | 0.00062695 | TKT/ASNS/ASS1/CTH/ENO3/GOT1/ARG1/CPS1/SDS/GPT2/OTC/ALDOB | 12 |
| hsa00250 | Alanine, aspartate and glutamate metabolism | 8/329 | 37/8101 | 9.34E-05 | 0.00150025 | 0.0012896 | ASNS/AGXT2/ABAT/ASS1/GLS2/GOT1/CPS1/GPT2 | 8 |
| hsa00591 | Linoleic acid metabolism | 7/329 | 29/8101 | 0.00012315 | 0.00187311 | 0.0016101 | CYP1A2/CYP3A4/CYP2C9/CYP2C19/CYP2J2/CYP2C8/CYP2E1 | 7 |
| hsa00340 | Histidine metabolism | 6/329 | 22/8101 | 0.0001841 | 0.00266021 | 0.00228668 | ALDH3A1/CNDP1/HAL/ALDH2/AMDHD1/FTCD | 6 |
| hsa03320 | PPAR signaling pathway | 11/329 | 76/8101 | 0.00022537 | 0.00310147 | 0.00266599 | ACSL1/ACADM/CPT2/PLIN2/PCK1/SLC27A5/SLC27A2/ANGPTL4/EHHADH/APOA5/CYP8B1 | 11 |
| hsa04976 | Bile secretion | 12/329 | 90/8101 | 0.00026042 | 0.00342092 | 0.00294059 | SLC51B/SLCO1B3/SLCO1B1/KCNN2/CYP3A4/CA2/LDLR/SLC22A1/SLC27A5/ADCY1/SLC10A1/ABCB4 | 12 |
| hsa00360 | Phenylalanine metabolism | 5/329 | 17/8101 | 0.00044283 | 0.00534007 | 0.00459026 | ALDH3A1/GLYAT/TAT/GOT1/HPD | 5 |
| hsa04146 | Peroxisome | 11/329 | 82/8101 | 0.00044347 | 0.00534007 | 0.00459026 | MPV17/ACSL1/HAO1/EPHX2/HAO2/XDH/MLYCD/CAT/SLC27A2/EHHADH/PXMP2 | 11 |
| hsa04110 | Cell cycle | 14/329 | 124/8101 | 0.0004788 | 0.0055349 | 0.00475774 | PTTG1/CDC20/CCNB2/MCM4/MCM2/MCM6/MCM5/CCNA2/MCM7/MCM3/SFN/GADD45G/MYC/GADD45B | 14 |
| hsa00140 | Steroid hormone biosynthesis | 9/329 | 61/8101 | 0.00072277 | 0.00803388 | 0.00690583 | AKR1C3/SRD5A2/CYP1A2/CYP3A4/HSD17B2/CYP3A5/CYP2E1/HSD11B1/HSD17B6 | 9 |
| hsa00650 | Butanoate metabolism | 6/329 | 28/8101 | 0.0007564 | 0.0080963 | 0.00695948 | ABAT/ACSM3/ACADS/ACAT1/EHHADH/ACSM5 | 6 |
| hsa00330 | Arginine and proline metabolism | 8/329 | 51/8101 | 0.00093921 | 0.00969403 | 0.00833287 | CNDP1/HOGA1/SAT1/ALDH2/OAT/GOT1/ARG1/PRODH2 | 8 |
| hsa00030 | Pentose phosphate pathway | 6/329 | 30/8101 | 0.00111322 | 0.01109386 | 0.00953615 | TKT/G6PD/IDNK/FBP1/RGN/ALDOB | 6 |
| hsa00640 | Propanoate metabolism | 6/329 | 34/8101 | 0.00219934 | 0.02118701 | 0.01821211 | ABAT/ACADS/ACAT1/MLYCD/ALDH6A1/EHHADH | 6 |
| hsa04115 | p53 signaling pathway | 9/329 | 73/8101 | 0.00264159 | 0.02462644 | 0.0211686 | CCNB2/TP53I3/SFN/IGFBP3/GADD45G/THBS1/GADD45B/STEAP3/SERPINE1 | 9 |
| hsa00310 | Lysine degradation | 8/329 | 63/8101 | 0.0037469 | 0.03224325 | 0.02771592 | SETDB1/PLOD3/AADAT/GCDH/BBOX1/ALDH2/ACAT1/EHHADH | 8 |
| hsa00270 | Cysteine and methionine metabolism | 7/329 | 50/8101 | 0.00379332 | 0.03224325 | 0.02771592 | AGXT2/CTH/TAT/CDO1/GOT1/SDS/BHMT | 7 |
| hsa04979 | Cholesterol metabolism | 7/329 | 50/8101 | 0.00379332 | 0.03224325 | 0.02771592 | SORT1/CETP/LCAT/LDLR/LIPC/ANGPTL4/LPA | 7 |
| hsa01240 | Biosynthesis of cofactors | 14/329 | 156/8101 | 0.00435475 | 0.03595783 | 0.03090894 | FLAD1/PPOX/NQO1/KMO/PANK1/NADK2/GCH1/IDO2/TDO2/ALDH2/RGN/HPD/ALPL/HSD17B6 | 14 |
