# Supplementary material for: Centromere protein N may be a novel malignant prognostic biomarker for hepatocellular carcinoma
Source: PeerJ. 2021 May 3;9:e11342. doi: 10.7717/peerj.11342 (PMC8101454; doi:10.7717/peerj.11342)
Supplement: Table S5 [file peerj-09-11342-s008.docx]

| Table S5. A Venn diagram of the overlapping genes between DMNC and MCC | | |
| --- | --- | --- |
| Names | Total | Elements |
| DMNC MCC | 22 | KIF20A CDKN3 GINS2 NCAPG CENPF NUSAP1 MELK ASPM MCM2 FOXM1 OIP5 HMMR TRIP13 KIF4A RACGAP1 CENPN PTTG1 HJURP PRC1 CDCA8 KIF2C CENPM |
| MCC | 8 | AURKB AURKA TOP2A UBE2C CCNA2 CCNB2 CDC20 TPX2 |
| DMNC | 8 | NCAPD2 CDCA5 FEN1 MCM5 MCM6 MCM4 MCM7 UHRF1 |
