# Supplementary material for: Centromere protein N may be a novel malignant prognostic biomarker for hepatocellular carcinoma
Source: PeerJ. 2021 May 3;9:e11342. doi: 10.7717/peerj.11342 (PMC8101454; doi:10.7717/peerj.11342)
Supplement: Table S6 [file peerj-09-11342-s009.docx]

| Table S6. Module analysis of the protein-protein interaction network | | | | |
| --- | --- | --- | --- | --- |
| Module | Score | Nodes | Edges | Genes |
| 1 | 30.903 | 32 | 479 | HJURP; OIP5; CENPN; CENPM; NUSAP1; TRIP13; CDCA8; CDC20; TPX2; AURKA; GINS2; MCM4; NCAPG; AURKB; CDKN3; CCNB2; MCM2; PTTG1; CCNA2; KIF20A; RACGAP1; KIF2C; UBE2C; TOP2A; MELK; HMMR; FOXM1; KIF4A; CENPF; PRC1; CDCA5; ASPM |
| 2 | 12.000 | 12 | 66 | EVA1A; GPC3; APOA5; IGFBP1; STC2; SERPINA10; CP; SPP1; CKAP4; CYR61; IGFBP3; SPP2 |
| 3 | 6.857 | 8 | 24 | PON1; LPA; APOF; CETP; LDLR; LIPC; LCAT; HPR |
| 4 | 6.400 | 11 | 32 | CYP4F2; CYP2A6; CYP2E1; AOX1; CYP2J2; CYP2C8; CYP3A5; PTGS2; CYP4F3; CYP26A1; CYP2C19 |
| 5 | 6.250 | 9 | 25 | UHRF1MCM3;POLE2;FEN1;NCAPD2;MCM5;MCM7;KNTC1;MCM6 |
