# Supplementary material for: Centromere protein N may be a novel malignant prognostic biomarker for hepatocellular carcinoma
Source: PeerJ. 2021 May 3;9:e11342. doi: 10.7717/peerj.11342 (PMC8101454; doi:10.7717/peerj.11342)
Supplement: Table S7 [file peerj-09-11342-s010.docx]

| Table S7. Detailed information on the genes in the most significant module | | | |
| --- | --- | --- | --- |
| Clusters | Node Status | Score | Name |
| Cluster 0 | Clustered | 25.641 | HJURP |
| Cluster 0 | Clustered | 25.441 | OIP5 |
| Cluster 0 | Seed | 26.000 | CENPN |
| Cluster 0 | Clustered | 25.931 | CENPM |
| Cluster 0 | Clustered | 25.441 | NUSAP1 |
| Cluster 0 | Clustered | 25.441 | TRIP13 |
| Cluster 0 | Clustered | 25.441 | CDCA8 |
| Cluster 0 | Clustered | 25.441 | CDC20 |
| Cluster 0 | Clustered | 25.441 | TPX2 |
| Cluster 0 | Clustered | 25.441 | AURKA |
| Cluster 0 | Clustered | 25.931 | GINS2 |
| Cluster 0 | Clustered | 23.726 | MCM4 |
| Cluster 0 | Clustered | 25.441 | NCAPG |
| Cluster 0 | Clustered | 25.441 | AURKB |
| Cluster 0 | Clustered | 25.808 | CDKN3 |
| Cluster 0 | Clustered | 25.441 | CCNB2 |
| Cluster 0 | Clustered | 25.000 | MCM2 |
| Cluster 0 | Clustered | 25.441 | PTTG1 |
| Cluster 0 | Clustered | 25.441 | CCNA2 |
| Cluster 0 | Clustered | 25.441 | KIF20A |
| Cluster 0 | Clustered | 24.929 | RACGAP1 |
| Cluster 0 | Clustered | 25.441 | KIF2C |
| Cluster 0 | Clustered | 25.441 | UBE2C |
| Cluster 0 | Clustered | 25.441 | TOP2A |
| Cluster 0 | Clustered | 25.441 | MELK |
| Cluster 0 | Clustered | 25.808 | HMMR |
| Cluster 0 | Clustered | 25.744 | FOXM1 |
| Cluster 0 | Clustered | 25.441 | KIF4A |
| Cluster 0 | Clustered | 25.641 | CENPF |
| Cluster 0 | Clustered | 25.441 | PRC1 |
| Cluster 0 | Clustered | 23.000 | CDCA5 |
| Cluster 0 | Clustered | 25.441 | ASPM |
